# Linear Immunoglobulin A Dermatosis: A Rare Case Illustrating Successful Treatment With Dapsone

**DOI:** 10.7759/cureus.35569

**Published:** 2023-02-28

**Authors:** Joana Machado Morais, Mariana Meneses, Catarina Freitas, Herberto Oliveira, Inês Leite

**Affiliations:** 1 Serviço de Pediatria, Hospital Pedro Hispano, Porto, PRT; 2 Serviço de Anatomia Patológica, Hospital Pedro Hispano, Porto, PRT; 3 Serviço de Dermatologia, Hospital Pedro Hispano, Porto, PRT

**Keywords:** autoimmune bullous dermatosis, dapsone, diffuse rash, direct immunofluorescence, linear iga bullous dermatosis

## Abstract

This is the case report of a previously healthy four-year-old girl with a history of upper airway infection that was treated with a β-lactam antibiotic. She was seen in the emergency department one month later with vesiculobullous lesions with clear content that were isolated or grouped in rosettes. Direct immunofluorescence showed baseline linear positivity for immunoglobulin A (IgA) (+) and fibrinogen-positive bullous content with absent remaining immunosera expression. The observed results were compatible with linear IgA bullous dermatosis. After confirming the diagnosis and excluding glucose-6-phosphate dehydrogenase (G6PD) deficiency, dapsone was added to the initial treatment with systemic and topical corticosteroids. This case report is a reminder of the importance of a high index of clinical suspicion for this condition to reach a timely diagnosis.

## Introduction

Linear IgA bullous dermatosis (LABD) is considered by several studies as the most frequent autoimmune bullous dermatosis in children [1]. LABD is a rare autoimmune, idiopathic, or drug-induced dermatosis characterized by subepidermal deposition of immunoglobulin A (IgA) antibodies [2]. It occurs in both adults and children, with a global incidence of 0.5-2.3 cases/million/year [3]. The triggering causes most commonly described in the literature are drugs and infections, with the second being the most common in the pediatric population [4]. It is characterized by the onset of papules, tense vesicles, or blisters with serous or hemorrhagic content on normal or erythematous skin, arranged in an annular or herpetiform arrangement (“crown of jewels” or “pearl necklace”), and usually scattered over the abdomen, inguinal region, lower limbs, feet, hands, periorificial area, and face [2]. Definitive diagnosis is obtained through direct immunofluorescence [5,6]. Dapsone is the first-line treatment, with a rapid response [7,8]. Although it is usually well tolerated, it may be associated with severe adverse effects, requiring a comprehensive pre-treatment investigative study and regular surveillance of key serum markers [2,5,9]. We describe the case of a four-year-old child diagnosed with LABD who underwent treatment with dapsone and systemic and topical corticosteroids.

## Case presentation

**Figure 1:**
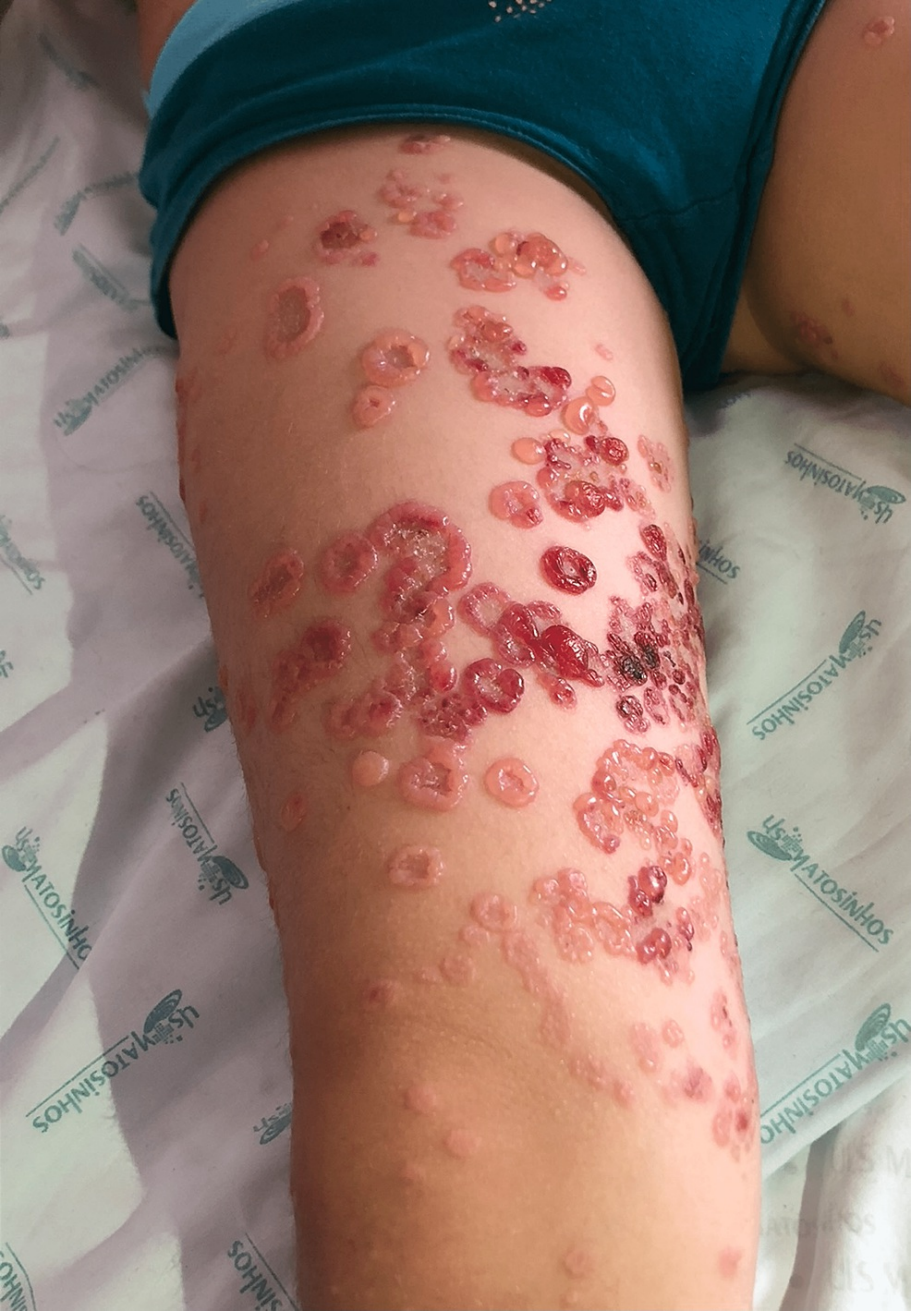
Clinical presentation Erythematous papules, vesiculobullous lesions with clear content, isolated or grouped in rosettes, and some lesions with signs of bacterial superinfection can be seen.

**Figure 2:**
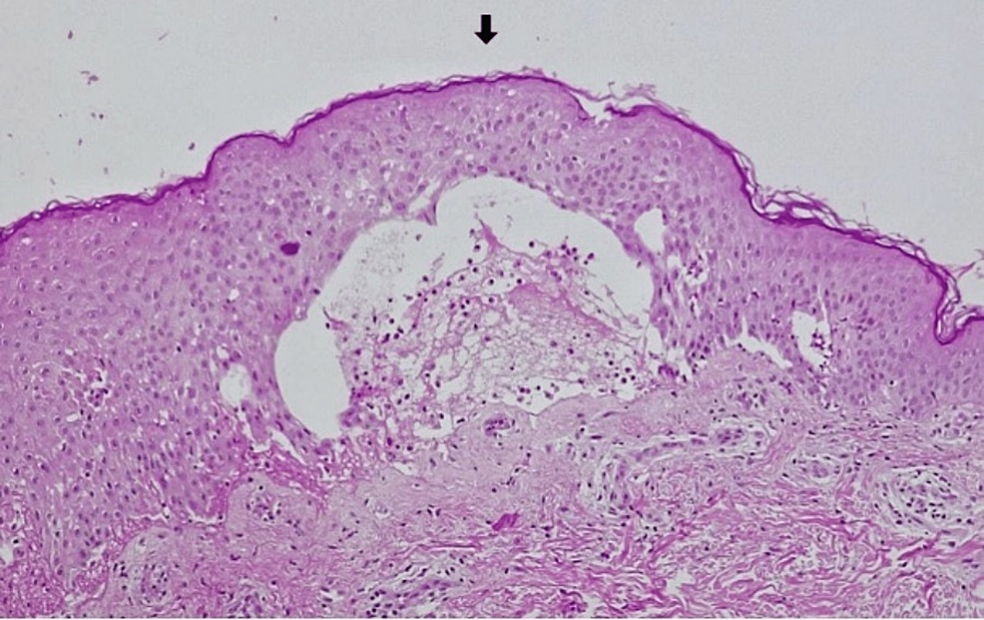
Histologic examination Skin with subepidermal blister with neutrophils, eosinophils, and fibrin (H&E, 100x).

**Figure 3:**
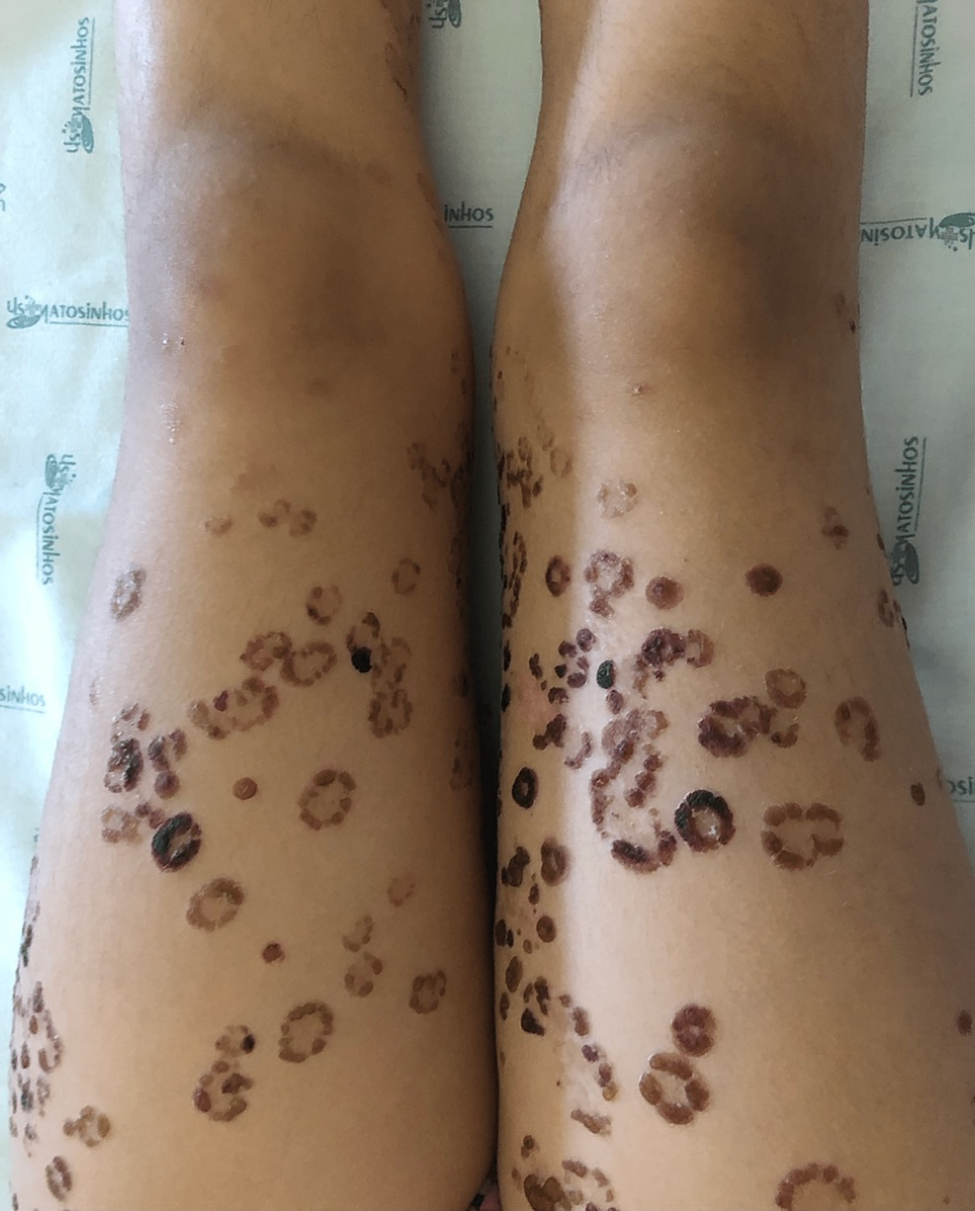
Clinical presentation Cicatricial phase on day 10 of hospitalization.

**Figure 4:**
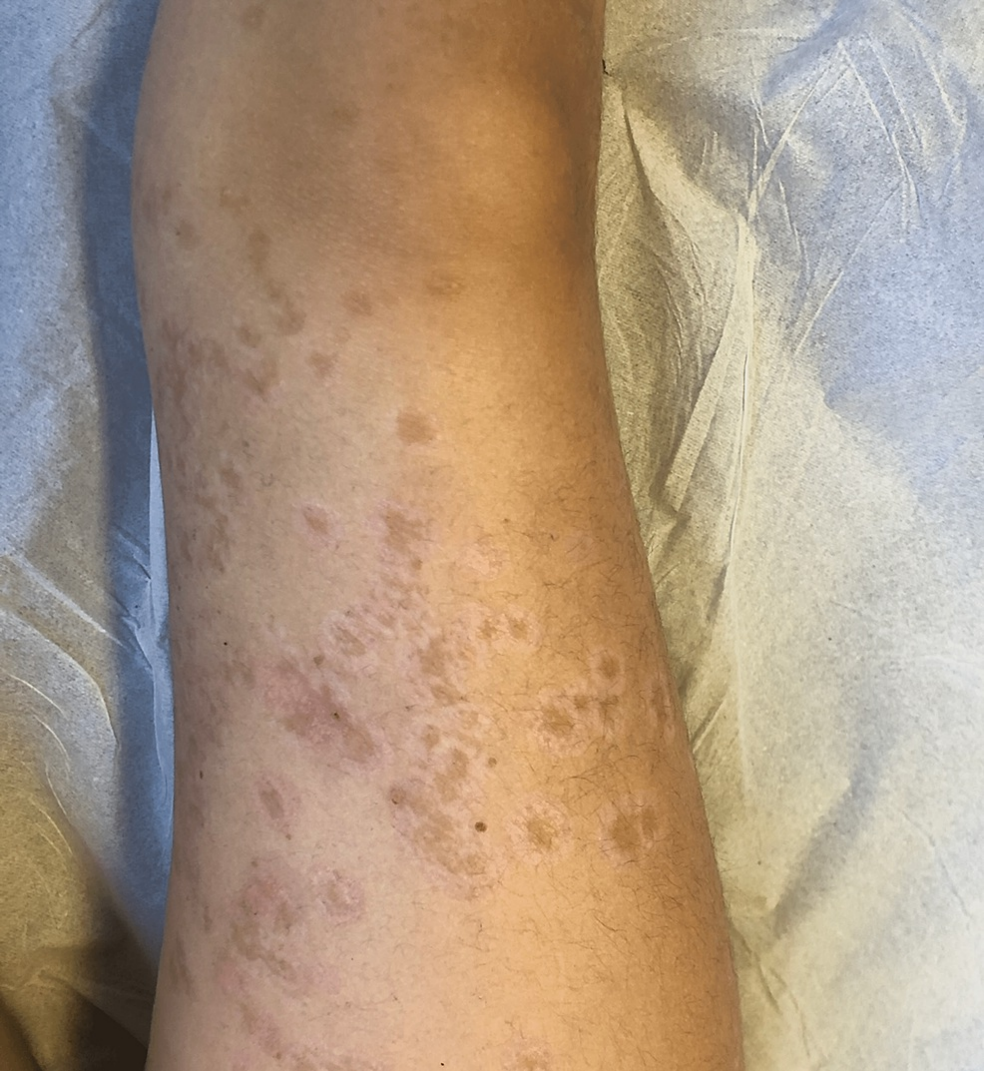
Clinical presentation Clinical reassessment two weeks after discharge.

The patient was a previously healthy four-year-old only child of non-consanguineous parents, with no history of hereditary family diseases. She presented to the emergency department with pruritic polymorphous exanthema (maculopapular and vesicular lesions progressing into blisters) scattered over the body that had been progressing for five days. The history around the time of this episode included acute tonsillitis, which was treated with oral amoxicillin for 10 days, and upper airway infection of probable viral etiology, which was treated with paracetamol and desloratadine. An objective examination performed in the emergency room showed exanthema scattered throughout the body, more exuberant in the lower limbs, with erythematous papules, vesiculobullous lesions (large and tense blisters) with clear content, isolated or grouped in rosettes, and some lesions with signs of bacterial superinfection (Figure 1). There were no obvious ophthalmological changes, but oral mucosa involvement was present. In the emergency service, she underwent an analytical study that revealed no abnormalities, in particular increased inflammatory parameters. Due to suspected skin infection, flucloxacillin 75 mg/kg/day and acyclovir 30 mg/kg/day (both intravenously) were administered. Considering the severity of the exanthema, the patient was hospitalized for etiological investigation and treatment. Topical corticosteroid therapy (betamethasone) was initiated on day 2 of hospitalization due to the hypothesis of LABD. On day 3, she was seen by the dermatologist, and a biopsy was performed. Histological examination of the lesion showed a spongiotic subepidermal blister containing neutrophils, eosinophils, and fibrin, slight perivascular lymphocytic infiltrate, and mild edema in the dermis (Figure 2). Direct immunofluorescence showed baseline linear positivity for IgA (+), fibrinogen-positive blister content, and absent remaining immunoserum (IgG, IgM, C3c, and C1q) expression. The findings were compatible with linear IgA dermatosis. After the negative result for herpes virus 1 and 2 serology and no bacterial growth on blood culture, therapy with acyclovir was suspended on day 3 and flucloxacillin on day 5 of hospitalization. On day 4 of hospitalization, prednisolone (1 mg/kg/day) was initiated. The exanthema progressed favorably after oral corticosteroid therapy was initiated, with partial regression of the lesion and control of pruritus with the administration of oral antihistamines. Dapsone was initiated on day 10 of hospitalization, after exclusion of G6PD deficiency. She was also seen in the Department of Ophthalmology, which ruled out eye involvement. As there was clinical improvement (Figure 3), with most lesions in the healing phase and the initiation of directed treatment, and no changes were found in the serum analysis (blood count, renal function, electrolytes, transaminase, and anti-transglutaminase antibody tests), she was discharged with a prescription of omeprazole, prednisolone 20 mg once daily, fluticasone propionate, dapsone 0.25 mg/kg once daily, cetirizine, and hydroxyzine. She was referred to a dermatologist, and regular clinical and analytical surveillance was maintained with weekly blood count in the first month of treatment, monthly blood count in the following four months, and biannually thereafter, as well as renal and liver function tests at the sixth month of treatment, and annually thereafter. The initial dose of dapsone was increased, maximum of 1-1.5 mg/kg/day, until adequate symptom control was achieved (Figure 4), but it always depended on analytical surveillance. If a dose was forgotten, the rash worsened, reflecting the good response to dapsone.

## Discussion

LABD, first described in the late 1970s as chronic bullous dermatosis of childhood, is a rare autoimmune, idiopathic, or drug-induced dermatosis characterized by subepidermal deposition of IgA antibodies [2]. This immune-mediated disease is considered in several studies to be the most frequent autoimmune bullous dermatosis in children [3]. The highest incidence is at preschool age, on average between 4 and 5 years of age, as in the case described above. It rarely occurs in the neonatal period [2,10]. There is no race or sex predilection [1,11]. IgA is deposited on the basement membrane and binds to the carboxyl-terminal of the same bullous pemphigoid (BP) antigen, BP180, forming immunopathological complexes. The IgA antibody is deposited on the basement membrane, specifically subclass A1, and induces the activation of the complement system and neutrophil chemotaxis, resulting in loss of dermo-epidermal junction adhesion. Antibody targets are classified into two groups: lamina lucida and sublamina densa. Both 97kDa and 120 kDa antigens, fragments of BP 180 localized in the dermal-epidermal junction, represent major antigenic targets of circulating IgA autoantibodies. [4]. Sublamina densa LABD is associated with the presence of 255 kDa and 290 kDa dermal antigens. Genetic associations with B8, DR3, DQ2, and Cw7 HLA (human leukocyte antigen.) haplotypes have been reported, where the presence of the first three is related to increased susceptibility for early onset of the disease, therefore being more associated with chronic bullous disease. Tumor necrosis factor 1 and 2 (TNF-1/2) genes are also related to the subtype of LABD, specifically with its duration, with TNF-1 representing a good prognostic factor associated with shorter disease duration. However, the reason underlying these phenomena is still unclear. Lymphocytes participate in the immune response, with a predominance of CD4+ T lymphocytes, neutrophils, and eosinophils. They promote tissue injury secondary to neutrophil recruitment, mast cell degranulation, and proteolytic enzyme release, leading to a local inflammatory process that results in blisters. The triggering causes most commonly described in the literature are drugs and infections, with the latter being the second most frequent in pediatric patients [4]. In adults, vancomycin is the main drug associated with LABD and may be manifested with a more severe presentation (e.g., toxic epidermal necrolysis). Our patient had been exposed to amoxicillin in the previous month, but it was not possible to identify a causal relationship. In drug exposure associated cases, the duration is only a few weeks, which did not characterize our patient’s illness [12]. The mechanism is unknown but may be associated with an immune response against a hapten-protein antigen derived from the drug. Linear IgA dermatosis can be associated with other autoimmune diseases such as inflammatory bowel disease, rheumatoid arthritis, psoriasis, systemic lupus erythematosus, and Sjogren’s syndrome [6]. Myelodysplastic syndromes and paroxysmal nocturnal hemoglobinuria have also been described in children with LABD [5,6]. Unlike dermatitis herpetiformis, LABD is not associated with coeliac disease [7]. Skin lesions have a different distribution in children compared to adults and are more frequent in the abdomen, inguinal region, inner thighs, anogenital region, feet, hands, and face [2]. They are characterized by the onset of tense papules, vesicles, or blisters with serous or hemorrhagic content on normal or erythematous skin, with an annular or herpetiform arrangement (“crown of jewels” or “pearl necklace”). New lesions usually appear on the periphery of lesions that are already resolving. Some studies refer to the presence of Nikolsky’s sign, which was not present in the patient [13]. Mucous membranes such as oral, ocular, and pharyngo-laryngeal mucosa may be affected [5]. The most frequently affected mucosa is the oral mucosa, with the onset of oral ulcers, as in our patient. Differential diagnoses include dermatitis herpetiformis, BP, and scabies. It is often confused with bullous impetigo [14]. The definitive diagnosis is obtained through direct immunofluorescence (DIF), which reveals IgA deposits linearly distributed along the basement membrane [2,5]. IgG, C3 component, and sometimes IgM deposits are also identified [5,6]. When it is not possible to perform DIF, indirect immunofluorescence (IIF) should be performed on serum samples. Using this technique, it is possible to detect the presence of circulating anti-basement membrane IgA antibodies in up to 75% of cases [15]. When the salt-split test is used, linear IgA deposits are detected on the roof of the blister (epidermal side) in lamina lucida LABD, whereas in sublamina densa LABD, deposits are detected on the blister floor (dermal side). The correlation between antibody titer and the degree of disease activity is still unknown. Despite the absence of randomized controlled studies confirming the efficacy of dapsone in both children and adults, several series and case studies describe rapid response and good tolerability, making dapsone the first-line treatment for LABD [7,8]. The initial dose ranges from 0.5 to 2 mg/kg/day and may be increased weekly until adequate symptom control is achieved [5,16]. Some studies suggest the co-administration of cimetidine, as it decreases dapsone-induced hematological toxicity [2]. Although usually well tolerated, it can be associated with serious adverse effects such as hemolysis, methemoglobinemia, agranulocytosis, hypersensitivity, and peripheral neuropathy. Therefore, before starting therapy, it is mandatory to exclude G6PD deficiency and perform an analytical study including a blood count with reticulocyte count and liver and kidney function tests [2,5,9]. Regular laboratory monitoring during treatment is also recommended. Topical corticosteroid monotherapy has only proven sufficient to control mild cases or localized disease and is usually used as adjuvant therapy. Systemic corticosteroids, such as prednisolone 0.5-1.0 mg/kg/day, help induce disease remission [5,6]. Second-line agents include sulphonamides, which are usually better tolerated by patients. Colchicine can be used in patients with G6PD deficiency [9,17]. The duration of treatment for idiopathic LABD varies, usually remaining for a few weeks after complete lesion resolution. Due to the possible adverse effects of first- and second-line treatments or in case systemic corticosteroids cannot be used, immunosuppressive agents such as azathioprine, mycophenolate mofetil, and cyclosporine are alternatives. They can also be used in patients with refractory disease [18]. As for prognosis, it is a benign disease, but with high impact on quality of life, anesthetic implications, and post-inflammatory hyper- and hypopigmentation. It may persist for months to years, subsequently presenting with spontaneous complete resolution, frequently before puberty.

## Conclusions

This case report describes the classic presentation of a rare disease, and LABD should be included in the differential diagnosis of any bullous dermatosis, especially in those with a history of viral infection or antibiotic therapy in close temporal proximity. The first-line treatment, dapsone, can be associated with serious adverse effects, and therefore regular clinical and investigative surveillance should be maintained. It is a benign and transient disease, but can occasionally persist for years, with high impact on quality of life and anesthetic implications.

## References

[1] Salman A, Tekin B, Yucelten D (2017). Autoimmune bullous disease in childhood. Indian J Dermatol.

[2] Fuentelsaz del Barrio V, Campos Domínguez M (2013). Dermatosis IgA lineal de la infancia. Pediatr Aten Primaria.

[3] Panelius J, Meri S (2015). Complement system in dermatological diseases - fire under the skin. Front Med (Lausanne).

[4] Jha P, Swanson K, Stromich J, Michalski BM, Olasz E (2017). A rare case of vancomycin-induced linear immunoglobulin A bullous dermatosis. Case Rep Dermatol Med.

[5] Fernandes KA, Galvis KH, Gomes AC, Nogueira OM, Felix PA, Vargas TJ (2016). Linear IgA and IgG bullous dermatosis. An Bras Dermatol.

[6] Lings K, Bygum A (2015). Linear IgA bullous dermatosis: a retrospective study of 23 patients in Denmark. Acta Derm Venereol.

[7] Fortuna G, Marinkovich MP (2012). Linear immunoglobulin A bullous dermatosis. Clin Dermatol.

[8] Ng SY, Venning VV (2011). Management of linear IgA disease. Dermatol Clin.

[9] Gil Sáenz FJ, Durán Urdániz G, Fernández Galar M, Ballester JG, Herrero Varasa A, Garcés Bordege R (2016). [Topical corticosteroids as a therapeutic alternative in linear immunoglobulin A bullous dermatosis in childhood: case report]. Arch Argent Pediatr.

[10] Zhao CY, Chiang YZ, Murrell DF (2016). Neonatal autoimmune blistering disease: a systematic review. Pediatr Dermatol.

[11] Mori F, Saretta F, Liotti L (2022). Linear immunoglobulin A bullous dermatosis in children. Front Pediatr.

[12] Navi D, Michael DJ, Fazel N (2006). Drug-induced linear IgA bullous dermatosis. Dermatol Online J.

[13] Pereira AR, Moura LH, Pinheiro JR, Pasin VP, Enokihara MM, Porro AM (2016). Vancomycin-associated linear IgA disease mimicking toxic epidermal necrolysis. An Bras Dermatol.

[14] Paloni G, Shardlow A, Maschio M, Berti I, Taddio A (2013). Clinical challenge a child with bullous skin lesions. JAMA Pediatr.

[15] Venning VA (2011). Linear IgA disease: clinical presentation, diagnosis, and pathogenesis. Dermatol Clin.

[16] Lara-Corrales I, Pope E (2010). Autoimmune blistering diseases in children. Semin Cutan Med Surg.

[17] Kenani N, Mebazaa A, Denguezli M, Ghariani N, Sriha B, Belajouza C, Nouira R (2009). Childhood linear IgA bullous dermatosis in Tunisia. Pediatr Dermatol.

[18] Farley-Li J, Mancini AJ (2003). Treatment of linear IgA bullous dermatosis of childhood with mycophenolate mofetil. Arch Dermatol.

